# Primary Hepatic Angiosarcoma Mimicking Multifocal Liver Abscess with Disseminated Intravascular Coagulation and Hemoperitoneum

**DOI:** 10.7759/cureus.1293

**Published:** 2017-05-30

**Authors:** Kyle Rowe, Fredy Nehme, Jacob Wallace, Travis McKenzie, Akash Joshi, William Salyers

**Affiliations:** 1 Internal Medicine, University of Kansas School of Medicine - Wichita; 2 Diagnostic Radiology, University of Kansas School of Medicine - Wichita

**Keywords:** angiosarcoma, disseminated intravascular coagulation, liver tumors, liver, liver abscess

## Abstract

Primary hepatic angiosarcoma (PHA), a rare and aggressive malignancy, has rarely been reported to present with disseminated intravascular coagulation with liver hemorrhage. Incidence is estimated at 0.5–2.5 cases per 10,000,000. To our knowledge, it has not been reported to mimic liver abscess with a septic presentation. Advanced imaging techniques may aid in the diagnosis, though biopsy with microscopy and immunohistochemistry is the mainstay. Prognosis is very poor, with a 5-year survival rate estimated at 6.4%. We present the case of a 70-year-old man who presented with sepsis thought to be due to a multifocal liver abscess, who did not respond to drainage and antibiotics. This atypical course led to further workup which subsequently revealed a diagnosis of PHA.

## Introduction

Primary hepatic angiosarcoma (PHA) is a very rare and highly aggressive malignancy, with an estimated incidence of 0.5–2.5 cases per 10,000,000 persons. An extremely vascular malignancy, PHA may rarely cause liver hemorrhage and intravascular coagulation abnormalities. It is known to have a very poor survival rate. To our knowledge, PHA has not been reported to present mimicking a liver abscess with a septic presentation. We present the case of a 70-year old man who presented with sepsis thought to be due to a multi-focal liver abscess diagnosed on computed tomography (CT). He did not respond to antibiotics with percutaneous drainage, and further workup performed resulted in the diagnosis of PHA.

## Case presentation

**Figure 1:**
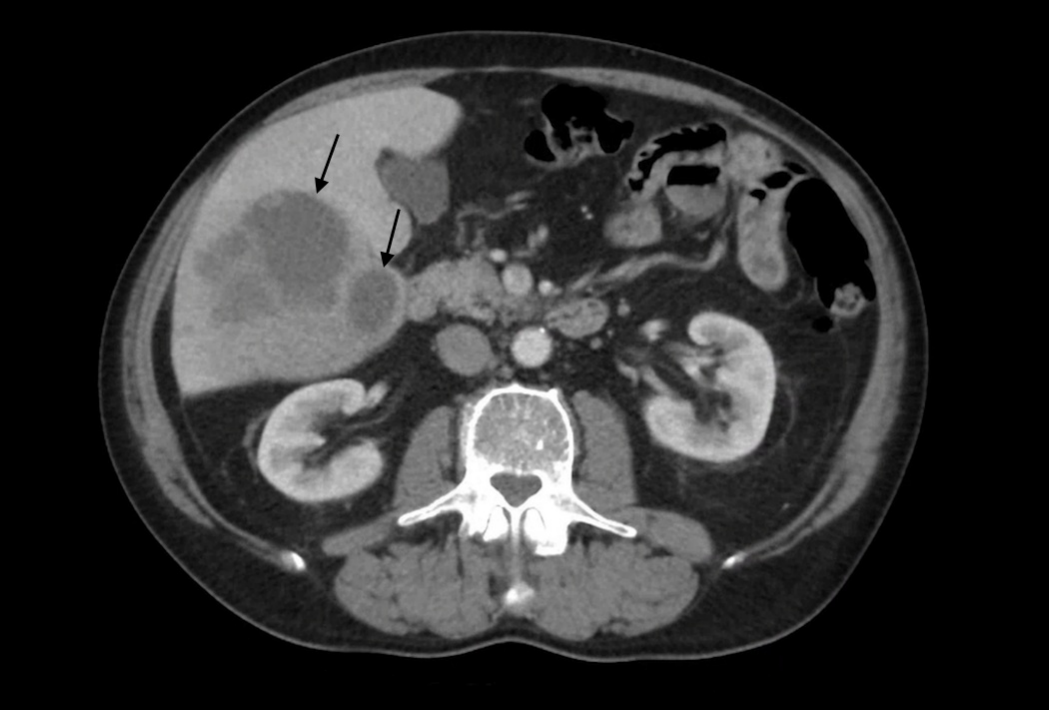
Contrast Enhanced Tomography of the Abdomen Contrast enhanced computed tomography of the abdomen performed on the day of presentation demonstrates focal areas of hypoattenuation within the right hepatic lobe (arrows) with subtle adjacent hypoattenuation predominantly in segment 6. Note the lack of perilesional hyperenhancement.

A 70-year-old Caucasian man presented to the emergency department with two days of fevers, chills, and abdominal fullness. His past medical history was significant for hypertension and fully excised rectal carcinoid tumor two years prior with subsequent negative metastatic workup. He had no recent travel history or immunocompromising conditions. He endorsed decreased appetite, fatigue, and minimal weight loss during the preceding two months, but otherwise had previously felt healthy. Physical examination was significant for fever of 39°C, tachycardia, tachypnea, and mild abdominal distention, but was otherwise unremarkable. Initial blood work showed leukocytosis of 19.9 x 10^3^ cells/ml with 89% neutrophils, hemoglobin of 11.8 g/dl, albumin of 2.8 g/dl, and alkaline phosphatase of 157 IU/l. There was no transaminitis, hyperbilirubinemia, coagulopathy, electrolyte disturbance, or acute kidney injury. Contrasted abdominal CT showed two fluid collections in the right lobe of the liver measuring up to 7 cm and 3 cm, with surrounding inflammatory changes (Figure 1). He was started on intravenous piperacillin/tazobactam and underwent placement of percutaneous drainage catheters to each fluid collection for source control.

**Figure 2:**
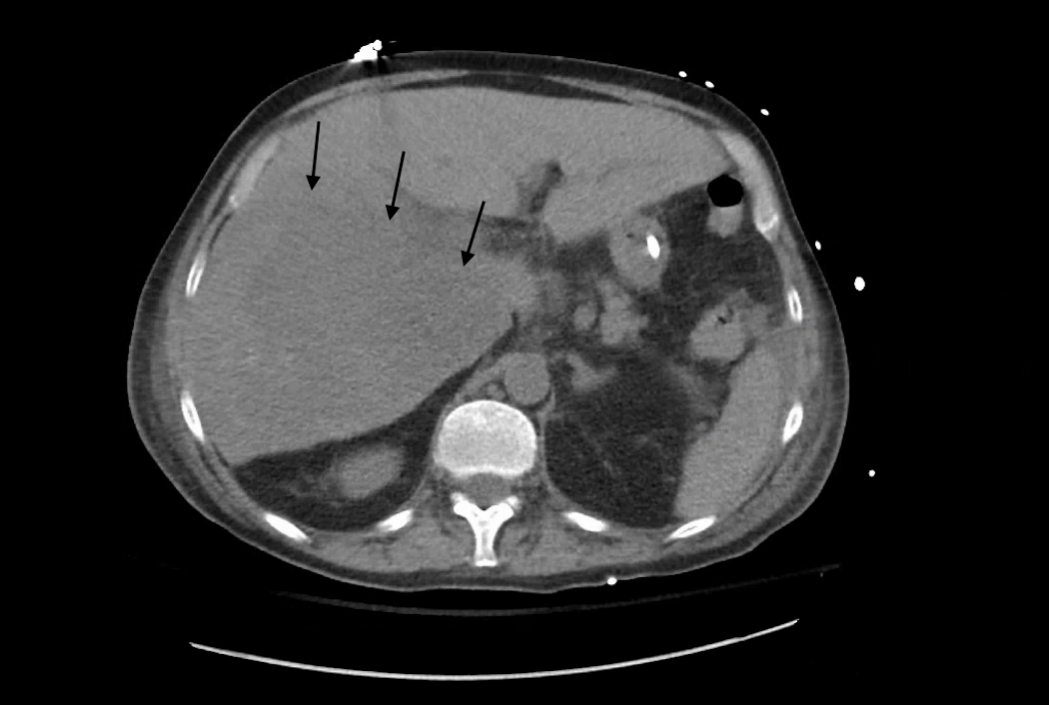
Non-Contrasted Computed Tomography of Abdomen Non-contrasted computed tomography of abdomen performed on hospital day 14 demonstrates increased area of hypoattenuation within the right hepatic lobe (arrows).

**Figure 3:**
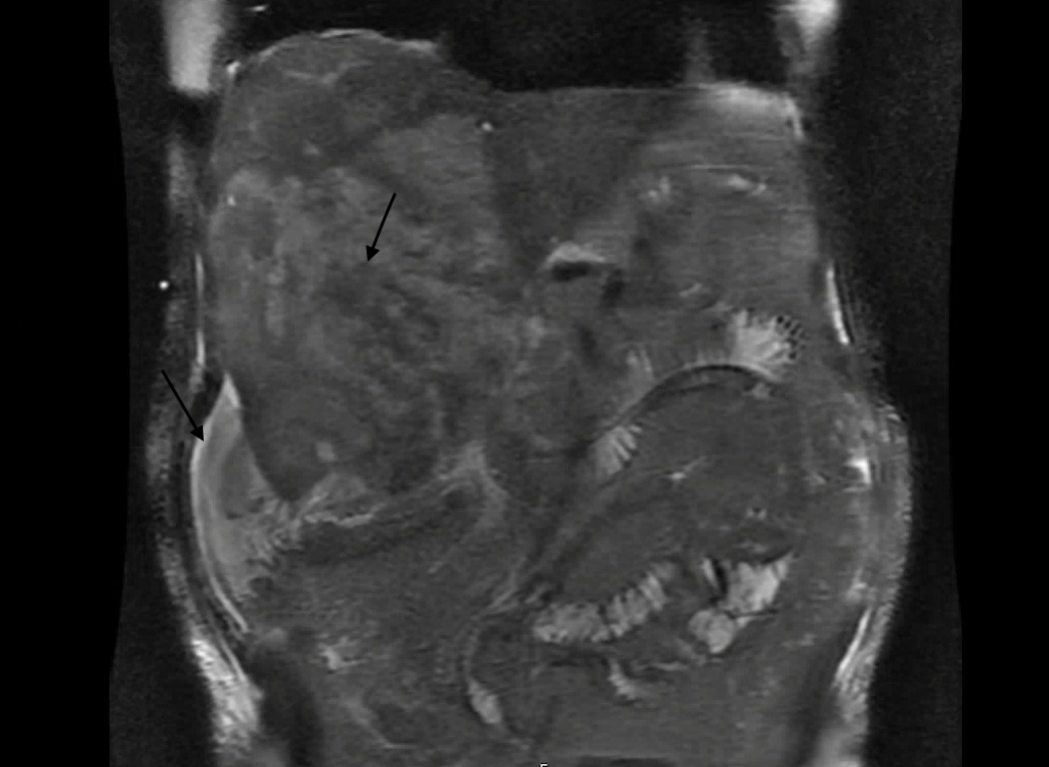
T2 Breath Hold Magnetic Resonance of Abdomen (Coronal) T2 weighted breath hold magnetic resonance of abdomen coronal view performed on hospital day 12 demonstrates diffuse T2 hyperintense signal through the right hepatic lobe with central areas of T2 hypointensity (arrow). Peri-hepatic ascites is also seen (arrow).

**Figure 4:**
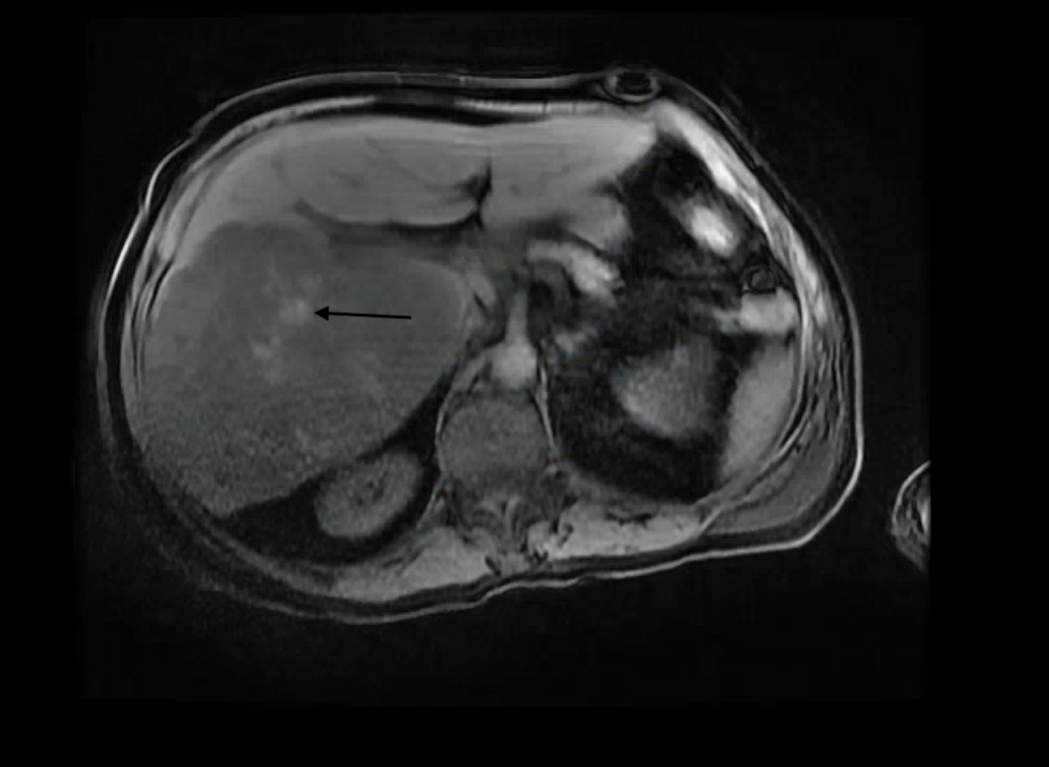
T1 Lava Flex Breath Hold Magnetic Resonance Imaging (Axial) T1 Lava Flex breath hold magnetic resonance imaging (MRI) of abdomen axial view demonstrates diffuse T1 hypointense signal through the right hepatic lobe with central areas of T1 hyperintensity (arrow). These findings represent central necrosis and hemorrhage.

A moderate amount of serosanguineous fluid was returned and sent for analysis which was negative for infection, including gram stain, culture, acid-fast, parasitic, and fungal examinations. Repeat abdominal CT demonstrated significant interval growth in the lesions (Figure 2). Magnetic resonance (MR) imaging of the liver further characterized this growth as containing central necrosis and hemorrhage (Figures 3-4). This further questioned the diagnosis of abscess and he underwent CT-guided liver biopsy. At this time, the patient began to develop hypotension, transaminitis, hyperbilirubinemia, acute kidney injury, worsening anemia, thrombocytopenia, and coagulopathy. Imaging demonstrated hemoperitoneum with evidence of hepatic source and the patient underwent embolization of the right hepatic artery in an attempt to control hemorrhage. D-Dimer was significantly elevated (40,942 ng/ml), with low fibrinogen (89 mg/dl), and schistocytes on peripheral smear consistent with disseminated intravascular coagulation (DIC), and he received multiple units of fresh frozen plasma in addition to platelets which reached a nadir of 14 k cells/ml. Alpha-fetoprotein and carcinoembryonic antigen were not elevated. CA-19-9 was significantly elevated at 943 U/ml. Peripheral flow cytometry was negative for malignancy.

**Figure 5:**
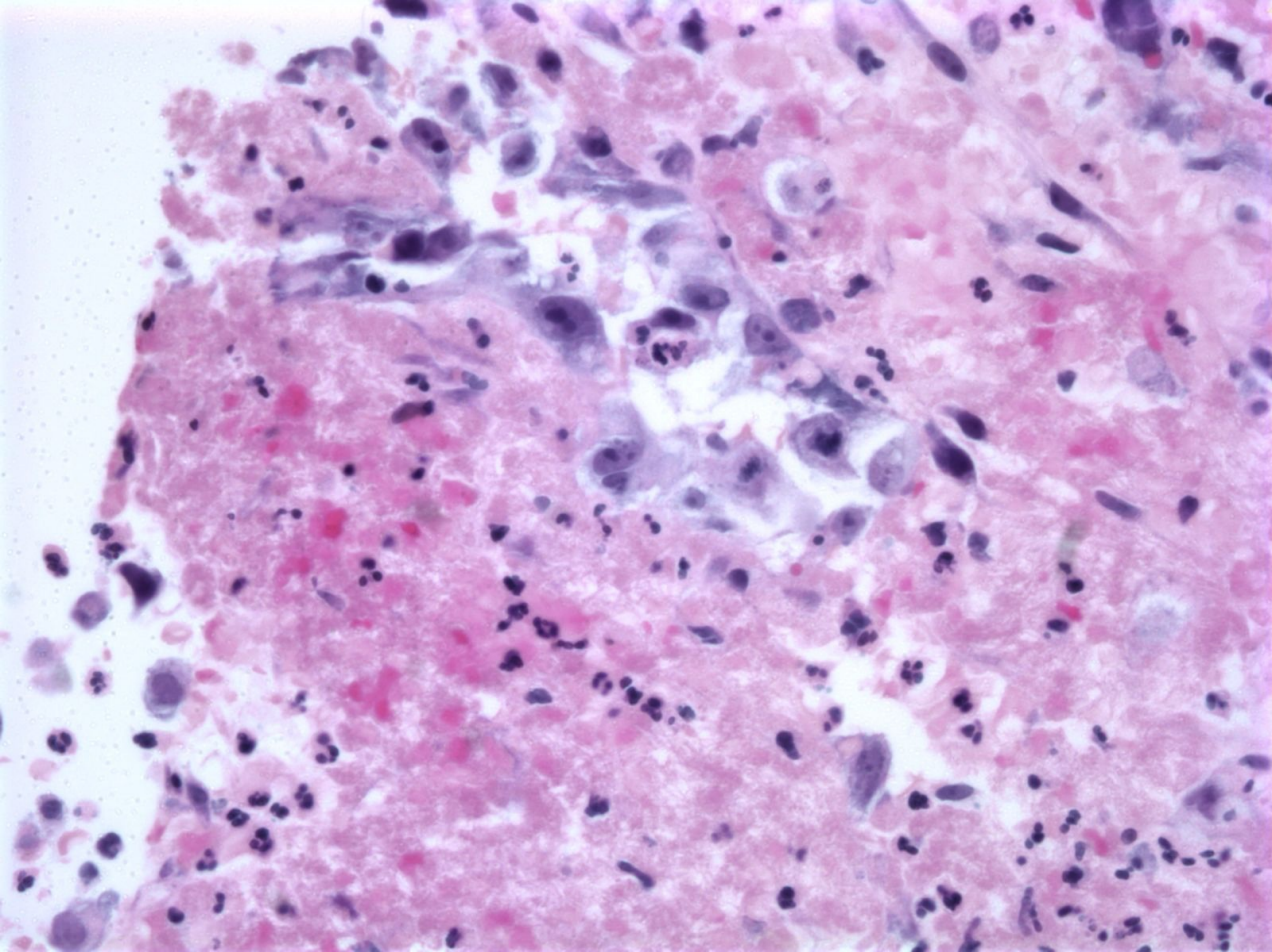
Hematoxylin and Eosin Stained Core Liver Biopsy (40x) Hematoxylin and eosin stained core liver biopsy (40x) demonstrating disrupted hepatic architecture with numerous highly atypical and pleomorphic cells within disrupted sinusoids, forming large anastomosing channels.

**Figure 6:**
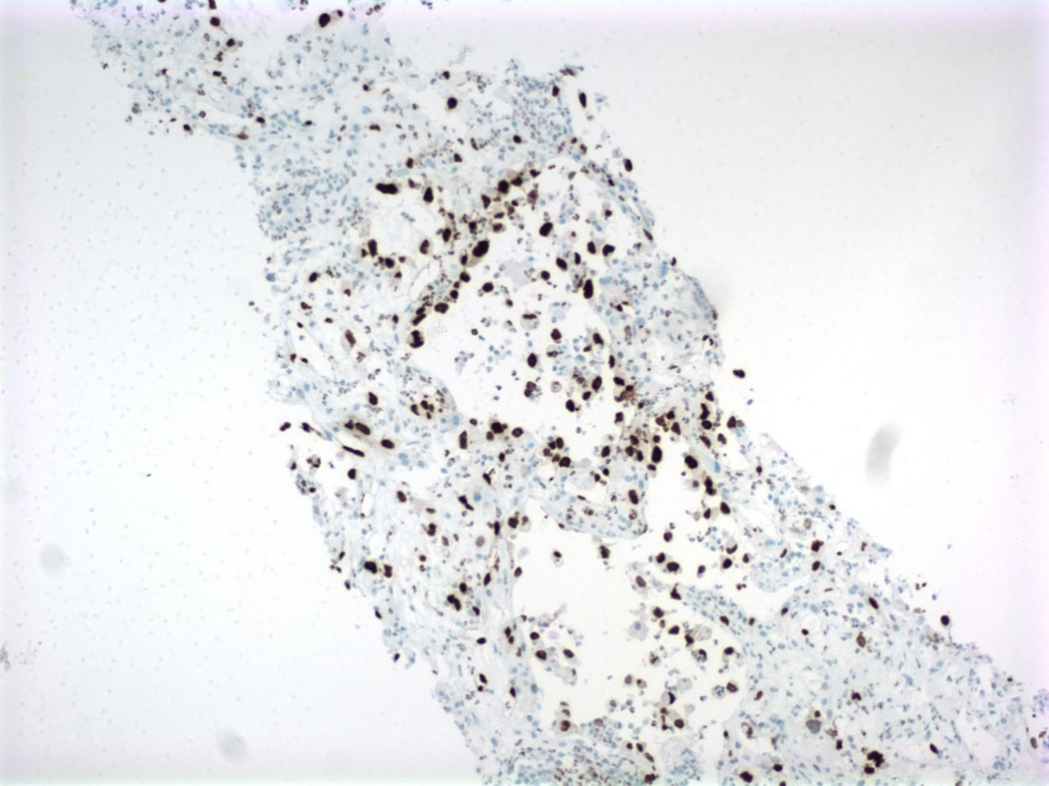
Immunohistochemistry of Liver Biopsy Highlighting Erythroblast-Transformation-Specific Related Gene (ERG) Positive Cells

**Figure 7:**
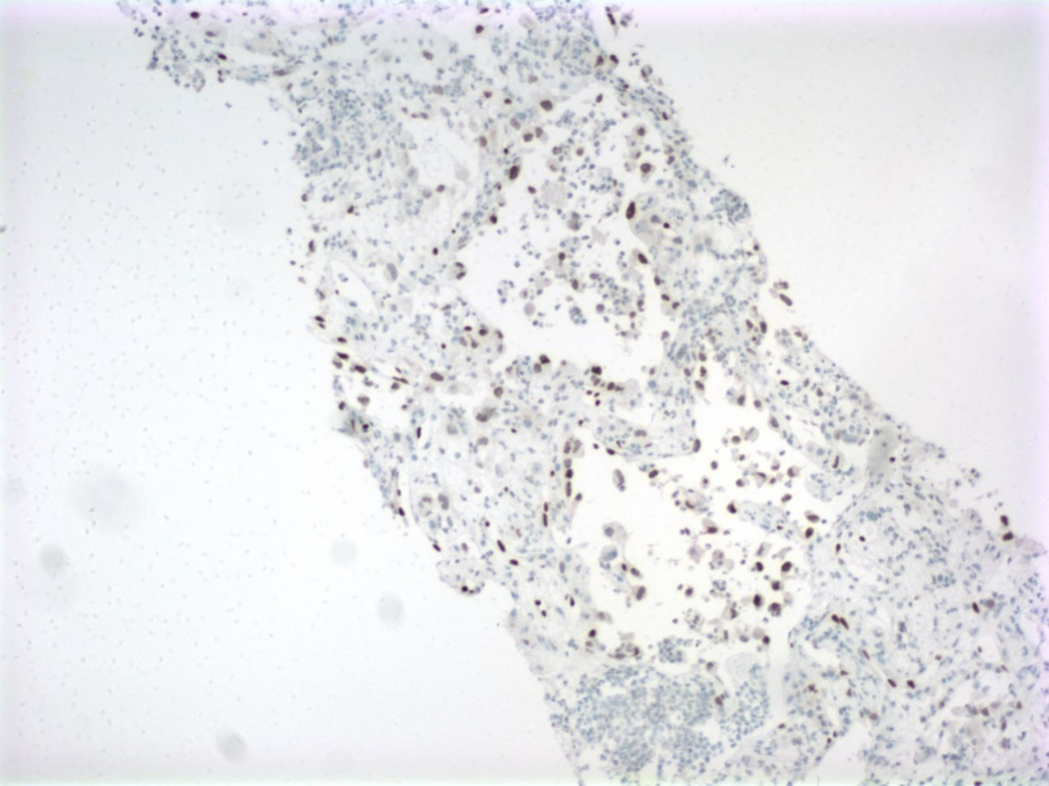
Immunohistochemistry (FLI-1)

Pathology from the liver biopsy showed marked neutrophilic infiltrate and necrosis with diffuse infiltration of highly atypical and pleomorphic cells within disrupted sinusoids, forming large anastomosing channels suspicious for angiosarcoma (Figure 5). Additional stains were positive for erythroblast-transformation-specific related gene (ERG) and FLI-1 consistent with the diagnosis of PHA (Figures 6-7). The patient’s status further deteriorated, with worsening encephalopathy and continued blood loss secondary to liver hemorrhage compounded by ongoing coagulopathy and thrombocytopenia. At the family’s request, further aggressive interventions were held and the patient passed away on hospital day 17. The family declined further post-mortem examination.

## Discussion

PHA is a rare and aggressive malignancy, estimated to occur at a rate of 0.5–2.5 cases per 10,000,000 persons, and accounts for 2% of all primary hepatic malignancies [1, 10]. It is known to present a significant diagnostic challenge given its rare occurrence, non-specific symptomatology, and challenging radiographic diagnosis. There have been few reports of PHA presenting as a cystic lesion [2], and it has not been reported to mimic abscess within the liver, presenting with significant leukocytosis and fever as our patient did.

A 2015 review of CT and MR findings of 35 patients with proven angiosarcoma noted multifocal involvement in all cases, as well as rapid interval size progression in 96% of cases [3]. Typical imaging features of lesions found in that study included: mean attenuation of 36 Hounsfield units (30-45) by CT, heterogeneous foci of hypervascular enhancement on late arterial phase, and continued expansion of these foci during the portal venous phase. This differentiates the lesion from hepatocellular carcinoma which typically will display delayed washout, also seen on MR [4]. The remainder of the lesion beyond these foci is comparable with blood attenuation, consistent with the known hemorrhagic nature of this tumor [3]. Hepatic peliosis, hemangioendothelioma, and angiomyolipoma are all included in the differential for these imaging findings. Abscess is also included in the differential, and clinical presentation should be incorporated into the decision-making, though malignancy can cause a similar presentation of fever and leukocytosis as in this case.

The presentation is highly variable and fulminant hepatic failure, disseminated intravascular coagulation, and fatal hemorrhage have all been reported [5-6]. Diagnosis hinges on imaging, which is non-specific, and biopsy which carries a significant risk of inducing hemorrhage. Microscopic examination shows spindle-shaped cells with both sinusoidal and solid patterns, and immunohistochemical staining positive for ERG, factor VIII, CD 31, and CD 34 [7, 10]. While CD31 has traditionally been a reliable marker, one study found ERG to be positive in 100% of samples, while CD34 was positive in 87.5% [10].

Ideally, treatment is complete resection, especially when the disease is limited to one segment, though many patients have multi-lobar involvement, metastases, or life-threatening complications on the presentation that preclude surgery [8]. Liver transplant is controversial and generally avoided as survival is poor and recurrence rates are high [9]. Alternative palliative therapies include transcatheter arterial chemoembolization or systemic chemotherapy, though there is only limited observational data to support efficacy.

Survival after PHA is very poor, with 5-year survival estimated at 6.4% [10]. To our knowledge, there is no known association between a history of gastrointestinal carcinoid tumors and subsequent development of angiosarcoma, as occurred in this patient, and there is no mechanistic explanation to suspect such an association.

## Conclusions

PHA is a rare and aggressive malignancy that presents a significant diagnostic challenge. The presentation is highly varied, and can include life-threatening manifestations including fatal hemorrhage, acute liver failure, and DIC. Though not typical, CA 19-9 may be significantly elevated. Treatment is surgical resection, especially if the lesion is limited to one lobe. Liver transplant is generally avoided. Prognosis remains very poor with estimated 5-year survival of 6.4%. This case highlights the rarity of the disease, difficulty of diagnosis, and the importance of early recognition when possible.
